# Supplementary material for: Testing the climate intervention potential of ocean afforestation using the Great Atlantic Sargassum Belt
Source: Nat Commun. 2021 May 7;12:2556. doi: 10.1038/s41467-021-22837-2 (PMC8105394; doi:10.1038/s41467-021-22837-2)
Supplement: Supplementary file 1 — Supplementary Information [file 41467_2021_22837_MOESM1_ESM.docx]

**Testing the climate intervention potential of ocean afforestation using the Great Atlantic *Sargassum* Belt**

Lennart T. Bach^1^*, Veronica Tamsitt^2,3^, Jim Gower^4^, Catriona L. Hurd^1^, John A. Raven^5,6,7^, Philip W. Boyd^1^

*Corresponding author: [Lennart.bach@utas.edu.au](mailto:Lennart.bach@utas.edu.au)

^1^Institute for Marine and Antarctic Studies, University of Tasmania, Hobart, Tasmania, Australia

^2^University of New South Wales, Sydney, Australia

^3^Centre for Southern Hemisphere Oceans Research, CSIRO Oceans and Atmosphere, Hobart, Tasmania, Australia

^4^Fisheries and Oceans Canada, North Saanich, BC, Canada

^5^Division of Plant Sciences, University of Dundee at the James Hutton Institute, Invergowrie, Dundee, DD2 5DA, UK

^6^Climate Change Cluster, University of Technology, Sydney, Ultimo, NSW 2006, Australia

^7^School of Biological Sciences, University of Western Australia, 35 Stirling Highway, Crawley, WA 6009, Australia

This document contains Supplementary Tables, Figures, and Discussions relevant for the analysis and understanding of the study.

Supplementary Table 1. *Sargassum* wet weight, particulate carbon, and dissolved organic carbon (DOC) during the 2018 Great Atlantic *Sargassum* Belt (Mt = Million tonnes). Wet weights in the left column are satellite-based data for 89°W-15°E, 5°S-25°N as reported by Wang et al.^1,2^. Wet weight build-up during growth season was 20.35-1.55 = 18.8 Mt. Total particulate carbon (TPC) was calculated by dividing the wet weight by the wet weight/TPC ratio of 18.41 from ref.^3^ Particulate organic carbon (POC), and particulate inorganic carbon (PIC) were calculated by multiplying TPC with the POC/TPC (0.792) and PIC/TPC (0.208) ratios, respectively. These ratios are based on 9.4 % of the *Sargassum* wet weight being CaCO_3_. The POC and PIC data provided here has been used in Fig. 1d in the main text. Dissolved organic carbon (DOC) production rates are shown in the last column. Cumulative production for this period was 1.07 Mt DOC.

| Month | Wet weight (Mt) | TPC (Mt) | POC (Mt) | PIC (Mt) | DOC production (Mt) |
| --- | --- | --- | --- | --- | --- |
| Nov-17 | 1.55 | 0.084 | 0.066 | 0.017 | 0.013 |
| Dec-17 | 2.91 | 0.158 | 0.125 | 0.033 | 0.026 |
| Jan-18 | 6.36 | 0.345 | 0.273 | 0.072 | 0.057 |
| Feb-18 | 10.08 | 0.547 | 0.434 | 0.114 | 0.081 |
| Mar-18 | 10.50 | 0.570 | 0.452 | 0.119 | 0.094 |
| Apr-18 | 12.36 | 0.671 | 0.532 | 0.140 | 0.107 |
| May-18 | 16.32 | 0.886 | 0.702 | 0.184 | 0.146 |
| Jun-18 | 20.35 | 1.105 | 0.875 | 0.230 | 0.176 |
| Jul-18 | 16.73 | 0.909 | 0.720 | 0.189 | 0.149 |
| Aug-18 | 9.83 | 0.534 | 0.423 | 0.111 | 0.088 |
| Sep-18 | 4.75 | 0.258 | 0.204 | 0.054 | 0.041 |
| Oct-18 | 3.02 | 0.164 | 0.130 | 0.034 | 0.027 |
| Nov-18 | 2.70 | 0.146 | 0.116 | 0.030 | 0.023 |
| Dec-18 | 4.37 | 0.237 | 0.188 | 0.049 | 0.039 |

Supplementary Table 2. Data used to calculate changes in radiative forcing due to the increase of albedo associated with seaweed farming in the oceans. *Sargassum* coverage data was reported by Wang et al.^1,2^. Incoming solar flux was obtained from the NASA Giovanni online tool (<https://giovanni.gsfc.nasa.gov/giovanni/>) as described in the methods. Also included are upper and lower bounds for decreases in radiative forcing assuming an increase in albedo (Δa) of 0.01 or 0.1. Bold numbers at the bottom are summed values for the entire growth cycle.

| **Year** | **Month** | ***Sargassum* coverage (km^2^)** | **Incoming solar flux (W/m^2^)** | **Decrease in radiative forcing for** Δa **= 0.01 (PJ/month)** | **Decrease in radiative forcing for** Δa **= 0.1 (PJ/month)** |
| --- | --- | --- | --- | --- | --- |
| 2017 | December | 870 | 215 | 4 | 40 |
| 2018 | January | 1903 | 245 | 10 | 100 |
| 2018 | February | 3018 | 280 | 16 | 164 |
| 2018 | March | 3145 | 280 | 19 | 189 |
| 2018 | April | 3702 | 280 | 21 | 215 |
| 2018 | May | 4887 | 280 | 29 | 293 |
| 2018 | June | 6093 | 245 | 31 | 310 |
| 2018 | July | 5010 | 215 | 23 | 231 |
| 2018 | August | 2945 | 215 | 14 | 136 |
| 2018 | September | 1423 | 215 | 6 | 63 |
| 2018 | October | 905 | 180 | 3 | 35 |
| 2018 | November | 807 | 215 | 4 | 36 |
|  |  |  |  | **181** | **1811** |

Supplementary Table 3. Decrease of radiative forcing for a range of albedo enhancements through ocean afforestation. An increase in albedo of 0.01 would constitute a moderate change, representative for afforestation deeper in the water column (e.g. 5 m below surface) whereas 0.1 would be a more representative for a dense canopy at the sea surface as is the case for *Sargassum*. The underlying scenario for a 6093 km^2^ seaweed farm in the tropics is detailed in the methods.

| **Increase in albedo** | **Decrease in radiative forcing (PJ/year)** |
| --- | --- |
| 0.01 | 181 |
| 0.02 | 362 |
| 0.03 | 543 |
| 0.04 | 724 |
| 0.05 | 905 |
| 0.06 | 1086 |
| 0.07 | 1267 |
| 0.08 | 1449 |
| 0.09 | 1630 |
| 0.1 | 1811 |


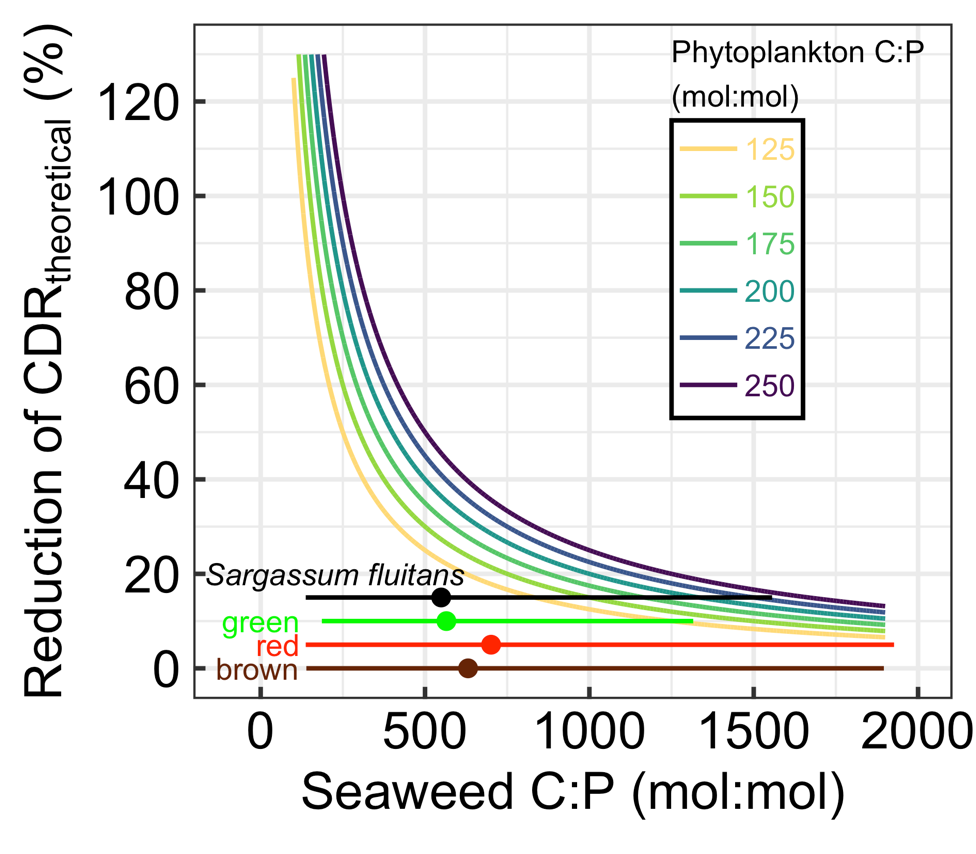


Supplementary Figure 1. Reduction of the theoretical CO_2_ removal potential (CDR_theoretical_) due to nutrient re-allocation, assuming P instead of N-limitation (results of a similar calculation with N-limitation are shown in Fig. 2b of the main text). As for N-limitation, the reduction of CDR_theoretical_ becomes more pronounced the more the seaweed C:P ratio approaches the phytoplankton C:P ratio. We used a range of phytoplankton C:P ratios^4^ as indicated by the color code. The horizontal lines display the range of C:N in *Sargassum*^5^ and green/red/brown seaweeds, respectively^6^ (the height of the lines on the y-axis has no meaning). The solid symbols on the horizontal lines are averages (*Sargassum* average = 550).


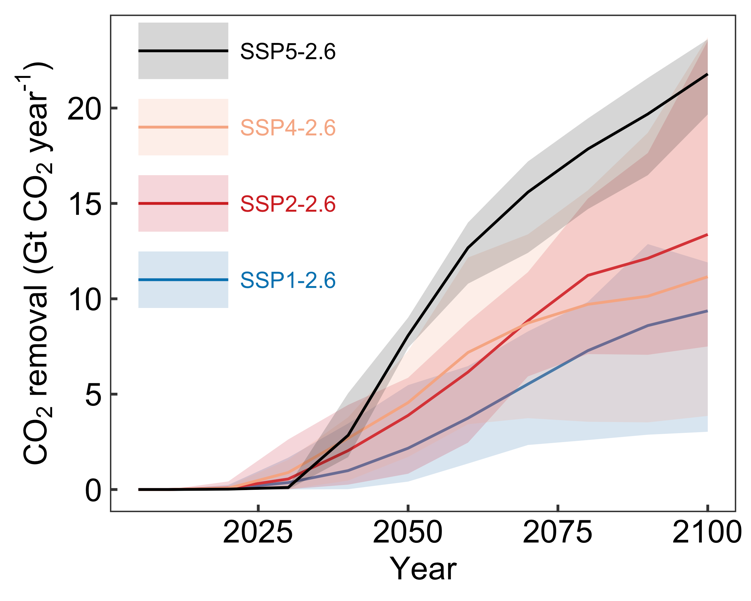


Supplementary Figure 2: The magnitude of CO_2_ removal included in runs of Integrated Assessment Models (IAMs) where global warming is below 2 °C at the end of the simulation (i.e., year 2100). The solid lines show the shared socioeconomic pathway (SSP) scenario means and the coloured shaded area the spread (minimum-maximum) of the different IAMs. Please note that there was no IAM simulation where global warming was below 2 °C for SSP3.


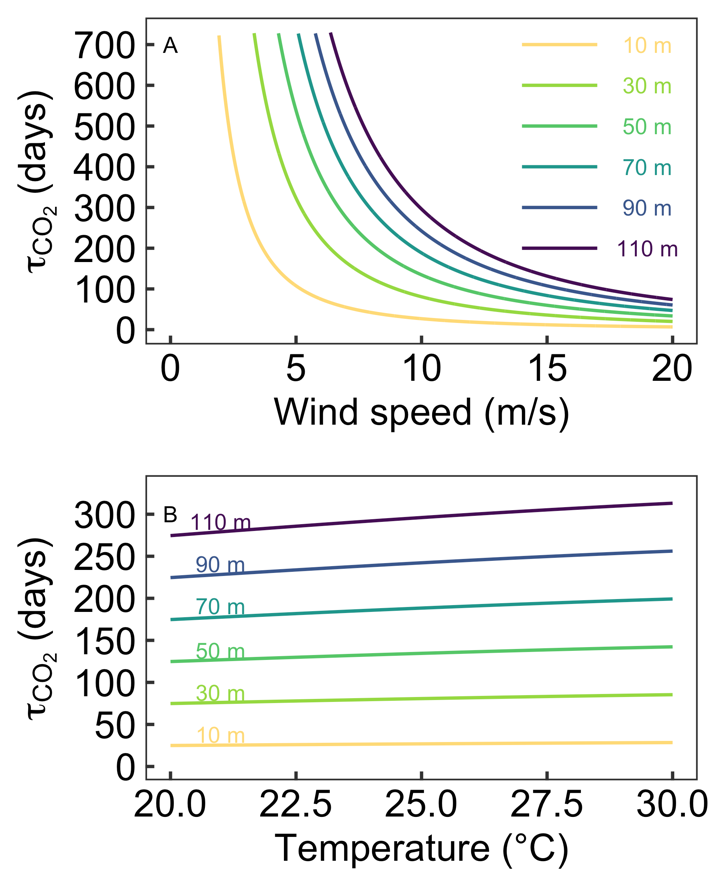


Supplementary Figure 3. The air-sea equilibration timescales of CO_2_ (𝜏_CO2_) under different environmental settings. (A) 𝜏_CO2_ as a function of wind speed for different mixed layer depths as indicated by the colour code. (B) 𝜏_CO2_ as a function of temperature for different mixed layer depths as indicated by the colour code.


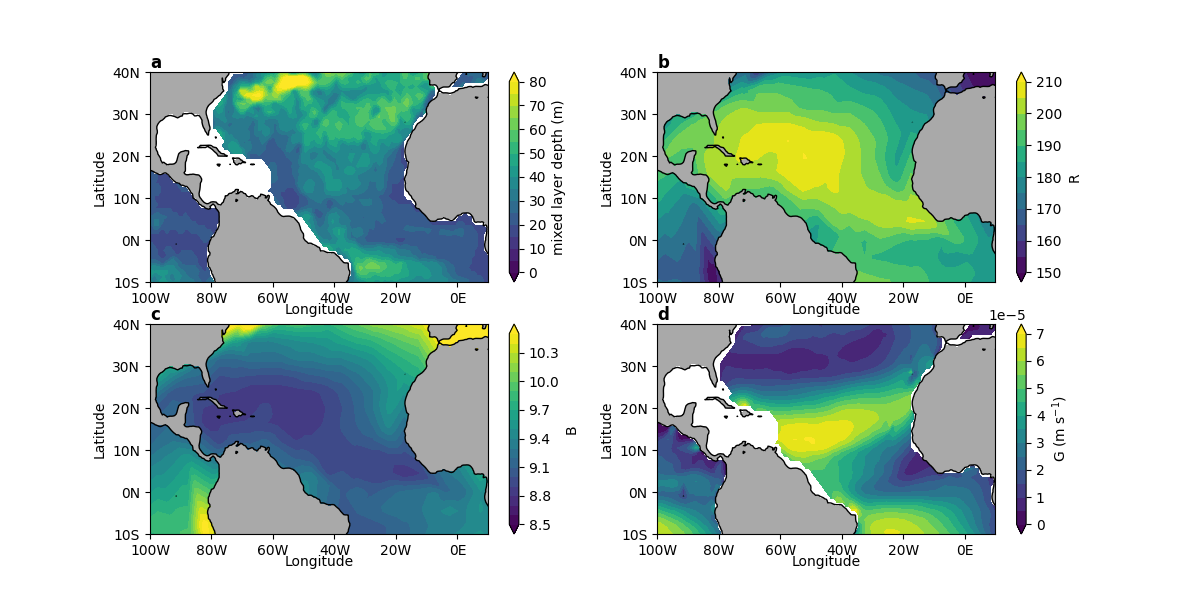


Supplementary Figure 4. Annual means of individual components in equation 4 (methods) which was used to calculate air-sea equilibration timescales of CO_2_ (𝜏_CO2_). (a) Mixed layer depth. (b) The ratio of dissolved inorganic carbon (DIC) to CO_2_ (R). (c) The Revelle factor (B). (d) The gas transfer velocity (G).


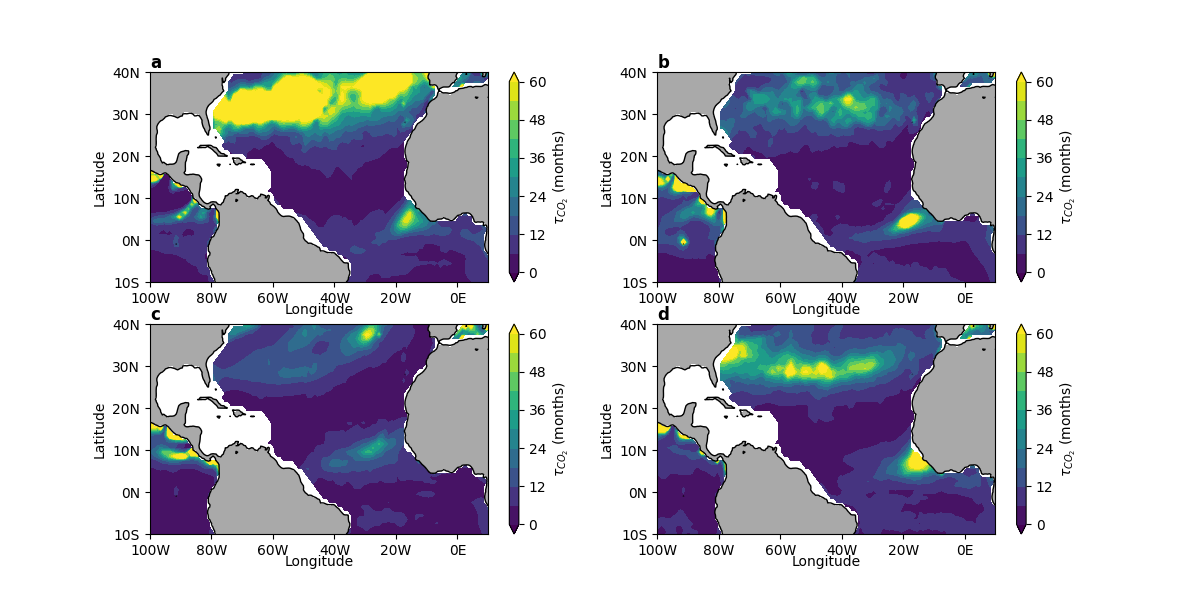


Supplementary Figure 5. Seasonal means of air-sea equilibration timescales of CO_2_ (𝜏_CO2_) in the GASB region. (a) December - February. (b) March – May. (c) June - August. (d) September – October.


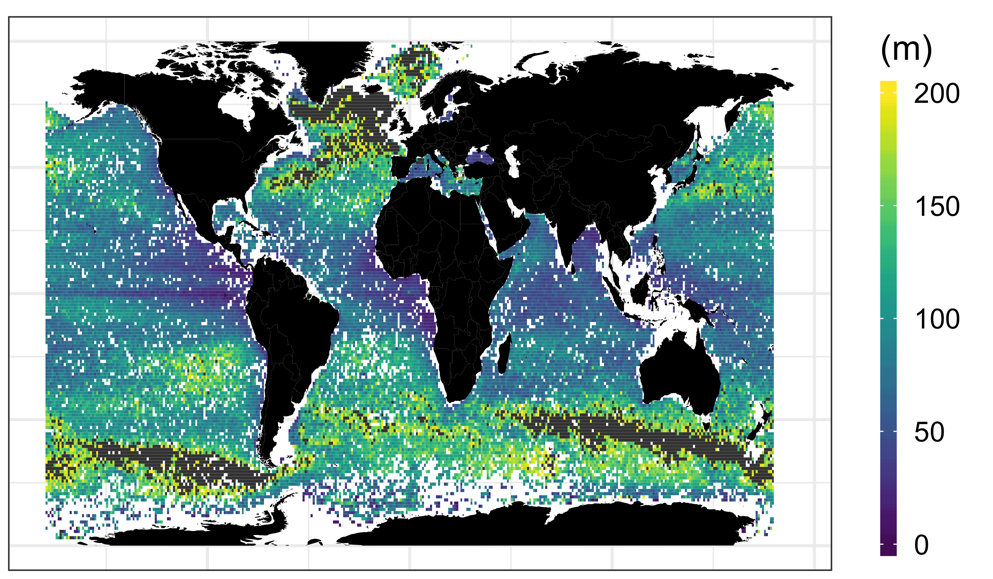


Supplementary Figure 6. Maximum monthly mean mixed layer depth based on an ARGO float climatology^7^. We used only grid fields where data from a complete seasonal cycle were available (i.e. complete from January to December). Dark-grey shaded ocean areas are mixed layer depths >200 m. Please note, monthly mean maxima generally occur in boreal winter in the Northern Hemisphere and austral winter in the Southern Hemisphere.

**Supplementary discussion 1: nutrient reallocation**

The degree of the CO_2_ removal (CDR) offset associated with nutrient reallocation depends on the phytoplankton C:N ratios and on the C:N of the seaweeds used for ocean afforestation. Phytoplankton C:N appear relatively constant at around 8 in the (sub)tropical Atlantic^8^ but seaweed C:N ratios vary with growth conditions and across taxa. Lower C:N values generally occur under nutrient-replete conditions that enable faster growth and higher biomass yields^5,9,10^. Thus, fertilizing and accelerating seaweed growth could reduce the C:N ratio and therefore reduce the CDR efficacy of ocean afforestation. For example, C:N ratios in holopelagic *Sargassum fluitans* can vary from 16-108 with the higher values generally found in more nutrient-limited offshore regions^5^. The corresponding CDR offset for such an intra-specific C:N range would be 7-50%. Benthic seaweeds, which are usually considered for ocean afforestation^11^, have C:N ratios of 6–78 mol:mol^6^. This is equivalent to a 13–130% reduction in CDR with higher percentages for the seaweed species with lower C:N ratios (Fig. 2B in the main text).

Application of external (non-marine) fertilizers to fuel ocean afforestation^12^, or recycling of nutrients from afforested seaweed biomass^11^, would eliminate the CDR offset related to nutrient reallocation, because the natural marine biological carbon pump would not be weakened. However, this would add investment/energetic/processing costs likely be associated with CO_2_ emissions, thereby potentially reducing CDR in other ways.

Ideas to utilize sub-surface nutrient pools and fuel afforestation by artificial upwelling^13^ may not ameliorate the CDR offset associated with nutrient re-allocation. This is because most sub-surface nutrients will be upwelled naturally at a later point, further downstream within ocean transports/currents, and then utilized by phytoplankton. How much later and how far downstream natural upwelling occurs depends on the depth from which the nutrients are sourced via artificial upwelling and the natural re-exposure times these nutrients would have had. Maximum mixed layer depths over the course of a season are generally shallower than 100 m in the GASB region (Supplementary Figure 6), indicating that nutrients from greater depths are less likely to be exposed to the surface in the same season. An ocean circulation inverse modelling study suggests that re-exposure times of subsurface waters to a depth of ~500 m is <100 years on a zonal average in the (sub)tropical Atlantic (ref.^14^), but timescales are likely to vary across regions and within this depth range. Accordingly, fuelling ocean afforestation by artificially upwelled nutrients will only shift the problem of nutrient re-allocation from phytoplankton to seaweeds in space and time. Nutrient reallocation, occasionally also referred to as nutrient robbing from downstream regions^15^, has already been raised as a problem for CDR with ocean iron fertilization^16–18^ and implicitly for afforestation^12^.

**Supplementary discussion 2: respiration of *Sargassum* biomass during sinking.**

Floatation (gas) bladders are morphological features of *Sargassum* and many other seaweed species, which make them positively buoyant^19,20^. Gas bladders can become non-functional over time through various natural processes such as grazing, calcite overgrowth, or collapse due to hydrostatic pressure^19,21^. However, for deliberately sequestering seaweeds on the seafloor it may be necessary to technically remove/destroy the gas bladders even though it is unclear how this could be achieved and if this would add CO_2_ emissions associated with the applied technology. If we assume that removal/destruction is possible, afforested *Sargassum* biomass would sink ~2500 m/d ^19,22^, reaching the deep ocean floor in much less than a week. Microbial respiration rates are too slow to respire large amounts of the sinking seaweed carbon within this timeframe^23^. Larger animals could potentially feed on sinking seaweeds and respire noticeable amounts. The few cases where small fragments of *Sargassum* was found in the stomachs of dolphinfish and tuna suggest that *Sargassum* was taken up accidentally while they were hunting for other fish^21^. Thus, there is no strong evidence for that pelagic fish contribute to the degradation of sinking seaweeds in the water column even though this would need to be confirmed in more targeted studies.

Recently, Ortega et al. used environmental DNA (eDNA) samples to trace the basin-scale distribution of macroalgae residuals/fragments and their occurrence down to 4000 m depth^24^. They reported that eDNA reads declined by 37.3 % km^-1^ depth and argued that a similar attenuation would also be present for the carbon associated with macroalgal eDNA^24^. As their study is based on suspended eDNA residuals/fragments from natural samples, it may not be perfectly representative for an ocean afforestation scenario where large amounts of complete seaweed thalli would sink rapidly into the deep ocean at one location due to the removal/destruction of the floatation bladders (see above). In this ocean afforestation scenario, we would expect a lower attenuation as the 37.3 % km^-1^ by Ortega et al.^24^, meaning that more of the seaweed carbon would arrive at the seafloor. Nevertheless, if we still assume that their estimated attenuation would also apply for *Sargassum* (or other seaweeds used for ocean afforestation), then much the sinking *Sargassum* POC would be respired and released into the water column as CO_2_ before reaching the seafloor.

POC respired higher in the water column would be re-exposed to the atmosphere much earlier than respired carbon locked in water masses close to the seafloor (e.g. ~0–400 years in the upper 1000 m of the (sub)tropical Atlantic in contrast to ≥700 years near the seafloor^14^). Thus, respiration of afforested seaweed biomass in the water column would reduce the permanence of CO_2_ in the oceans and therefore its economic value where permanence plays an important role^25,26^.

**Supplementary discussion 3: indirect albedo modifications**

Our albedo estimates consider changes in radiative forcing through direct albedo effects. They do not take indirect albedo modifications into account, which could be induced by seaweeds releasing various bioaerosols and other substances each influencing cloud albedo. In their 2018 review^27^, Brooks and Thornton emphasized that “the role of marine bioaerosols in cloud formation and climate is currently so uncertain that even the sign of the climate forcing is unclear”. Important bioaerosols and other climate-active substances linked to seaweeds comprise biogenic halocarbons or sulfur compounds^28–30^. In the following text we discuss dimethyl sulfide (DMS) as an illustrative example of how ocean afforestation could induce/alter indirect albedo effects.

Oxidation products of DMS contribute to aerosols and/or cloud condensation nuclei in the atmosphere, thereby inducing various climatic feedbacks^30,31^. Overall, these feedbacks have a significant cooling effect, with a globally averaged radiative forcing estimated between -1.13 to -2.43 W/m^2^ at the top of the atmosphere^30^. For comparison, CO_2_ has a warming effect of +1.82 W/m^2^ (ref.^52,63^). Dimethylsulfoniopropionate (DMSP), the precursor of DMS, is formed by seaweed and planktonic communities. We estimate that the particulate DMSP:N ratio by *Sargassum* could be between 0.00084-0.0013 mol:mol. This is based on the wet weight and N content of *Sargassum* from the GASB^3^ and DMSP contents of benthic (sub)tropical *Sargassum* ranging between 0.121-0.19 µmol DMSP g^-1^ wet weight^29,33^. The particulate DMSP:N estimated from planktonic communities in the subtropical North Atlantic near the Canary Islands is ~0.012 mol:mol^34,35^ and therefore >10 times higher than the *Sargassum* estimate. This suggests that planktonic communities in the subtropics produce more DMSP per limiting resource (i.e., N in the subtropical North Atlantic^36^) than *Sargassum* and that the nutrient reallocation from plankton towards *Sargassum* could reduce DMSP production.

The difference in DMSP:N between plankton and seaweeds will depend on the predominant phytoplankton and seaweed species within communities^29,37^. For example, DMSP contents of subtropical seaweeds from Hainan (China) differ 3-fold between species, from 0.01 to 14.7 µmol DMSP g^-1^ wet weight^29^. Hence, using other seaweeds than *Sargassum* for ocean afforestation may coincide with higher DMSP:N ratios in seaweed relative to planktonic communities. Furthermore, the positive correlation between DMSP and DMS observed in planktonic communities^38^, may not hold for ocean afforestation where large parts of the biomass must be sequestered before natural remineralization and some of the breakdown of DMSP to DMS occurs. Hence, DMS production from seaweeds may not be reliably inferred from DMSP precursor production. Finally, ocean afforestation would aggregate marine biomass and therefore DMSP/DMS production to afforested areas and reduce it elsewhere (due to nutrient re-allocation). Such local aggregations in DMSP/DMS production are potentially important, since DMS radiative forcing feedbacks vary regionally^30^. Altogether, these and potentially other unconstrained factors associated with ocean afforestation confound the assessment of an indirect albedo modification via the DMS feedback. Such complicating effects would likely also apply to other bioaerosol production again increasing uncertainties around estimation of the cumulative (i.e., direct and indirect) effects on albedo modification by *Sargassum*.

**References**

1. Wang, M., Hu, C. & Barnes, B. *Sargassum* density and coverage using Moderate Resolution Imaging Spectroradiometer (MODIS) satellite data from 2001-01-01 to 2018-12-31. *NOAA National Centers for Environmental Information* (2019). Available at: https://accession.nodc.noaa.gov/0190272. (Accessed: 25th March 2020)

2. Wang, M. *et al.* The great Atlantic *Sargassum* belt. *Science.* **364**, 83–87 (2019).

3. Wang, M. *et al.* Remote Sensing of *Sargassum* Biomass, Nutrients, and Pigments. *Geophys. Res. Lett.* **45**, 12,359-12,367 (2018).

4. Martiny, A. C. *et al.* Strong latitudinal patterns in the elemental ratios of marine plankton and organic matter. *Nat. Geosci.* **6**, 279–283 (2013).

5. Lapointe, B. E., West, L. E., Sutton, T. T. & Hu, C. Ryther revisited: Nutrient excretions by fishes enhance productivity of pelagic *Sargassum* in the western North Atlantic Ocean. *J. Exp. Mar. Bio. Ecol.* **458**, 46–56 (2014).

6. Atkinson, M. J. & Smith, S. V. C:N:P ratios of benthic marine plants. *Limnol. Oceanogr.* **28**, 568–574 (1983).

7. Holte, J., Talley, L. D., Gilson, J. & Roemmich, D. An Argo mixed layer climatology and database. *Geophys. Res. Lett.* **44**, 5618–5626 (2017).

8. Martiny, A. C., Vrugt, J. A., Primeau, F. W. & Lomas, M. W. Regional variation in the particulate organic carbon to nitrogen ratio in the surface ocean. *Global Biogeochem. Cycles* **27**, 723–731 (2013).

9. Lapointe, B. E. *et al.* Macroalgal blooms on southeast Florida coral reefs: I. Nutrient stoichiometry of the invasive green alga *Codium isthmocladum* in the wider Caribbean indicates nutrient enrichment. *Harmful Algae* **4**, 1092–1105 (2005).

10. Roleda, M. Y. & Hurd, C. L. Seaweed nutrient physiology: application of concepts to aquaculture and bioremediation. *Phycologia* **58**, 552–562 (2019).

11. N’Yeurt, A. D. R., Chynoweth, D. P., Capron, M. E., Stewart, J. R. & Hasan, M. A. Negative carbon via ocean afforestation. *Process Saf. Environ. Prot.* **90**, 467–474 (2012).

12. Orr, J. C. & Sarmiento, J. L. Potential of marine macroalgae as a sink for CO_2_: constraints from a 3-D general circulation model of the global ocean. *Water. Air. Soil Pollut.* **64**, 405–421 (1992).

13. Maruyama, S., Tsubaki, K., Taira, K. & Sakai, S. Artificial upwelling of deep seawater using the perpetual salt fountain for cultivation of ocean desert. *J. Oceanogr.* **60**, 563–568 (2004).

14. DeVries, T. & Holzer, M. Radiocarbon and Helium Isotope Constraints on Deep Ocean Ventilation and Mantle-^3^He Sources. *J. Geophys. Res. Ocean.* **124**, 3036–3057 (2019).

15. The Royal Society. *Geoengineering the climate: Science, Governance and Uncertainty Report No. RS1636*. (2009). doi:10.1007/s10098-010-0287-3

16. Gnanadesikan, A. & Marinov, I. Export is not enough: Nutrient cycling and carbon sequestration. *Mar. Ecol. Prog. Ser.* **364**, 289–294 (2008).

17. Oschlies, A., Koeve, W., Rickels, W. & Rehdanz, K. Side effects and accounting aspects of hypothetical large-scale Southern Ocean iron fertilization. *Biogeosciences* **7**, 4014–4035 (2010).

18. Rohr, T. Southern Ocean Iron Fertilization: An Argument Against Commercialization but for Continued Research Amidst Lingering Uncertainty. *J. Sci. Policy Gov.* **15**, (2019).

19. Johnson, D. L. & Richardson, P. L. On the wind-induced sinking of *Sargassum*. *J. Exp. Mar. Bio. Ecol.* **28**, 255–267 (1977).

20. Krause-Jensen, D. & Duarte, C. M. Substantial role of macroalgae in marine carbon sequestration. *Nat. Geosci.* **9**, 737–742 (2016).

21. Coston-Clements, L., Settle, L. R., Hoss, D. E. & Cross, F. A. *Utilization of the Sargassum habitat by marine invertebrates and vertebrates - a review*. *NOAA Technical Memorandum NMFS-SEFSC-296* (1991).

22. Baker, P. *et al.* Potential contribution of surface-dwelling *Sargassum* algae to deep-sea ecosystems in the southern North Atlantic. *Deep. Res. Part II Top. Stud. Oceanogr.* **148**, 21–34 (2018).

23. Bach, L. T. *et al.* The influence of plankton community structure on sinking velocity and remineralization rate of marine aggregates. *Global Biogeochem. Cycles* 2019GB006256 (2019). doi:10.1029/2019GB006256

24. Ortega, A. *et al.* Important contribution of macroalgae to oceanic carbon sequestration. *Nat. Geosci.* **12**, 748–754 (2019).

25. Herzog, H., Caldeira, K. & Reilly, J. An issue of permanence: Assessing the effectiveness of temporary carbon storage. *Clim. Change* **59**, 293–310 (2003).

26. Ruseva, T. *et al.* Rethinking standards of permanence for terrestrial and coastal carbon: implications for governance and sustainability. *Curr. Opin. Environ. Sustain.* **45**, 69–77 (2020).

27. Brooks, S. D. & Thornton, D. C. O. Marine aerosols and clouds. *Ann. Rev. Mar. Sci.* **10**, 289–313 (2018).

28. Leedham, E. C. *et al.* Emission of atmospherically significant halocarbons by naturally occurring and farmed tropical macroalgae. *Biogeosciences* **10**, 3615–3633 (2013).

29. Bischoff, B. *et al.* Preliminary assessment of the b-dimethylsulfoniopropionate (DMSP) content of macroalgae from the tropical island of hainan (People’s Republic of China). *Mar. Freshw. Res.* **45**, 1329–1336 (1994).

30. Fiddes, S. L., Woodhouse, M. T., Nicholls, Z., Lane, T. P. & Schofield, R. Cloud, precipitation and radiation responses to large perturbations in global dimethyl sulfide. *Atmos. Chem. Phys.* **18**, 10177–10198 (2018).

31. Hopkins, F. E. *et al.* The impacts of ocean acidification on marine trace gases and the implications for atmospheric chemistry and climate. *Proc. R. Soc. A Math. Phys. Eng. Sci.* **476**, (2020).

32. Myhre, G. *et al.* Anthropogenic and natural radiative forcing. in *Climate Change 2013: The Physical Science Basis. Contribution of Working Group I to the Fifth Assessment Report of the Intergovernmental Panel of Climate Change* (eds. Stocker, T. F. et al.) 658–740 (Cambridge University Press, 2013). doi:0.1017/CBO9781107415324.018

33. Broadbent, A. D., Jones, G. B. & Jones, R. J. DMSP in corals and benthic algae from the Great Barrier Reef. *Estuar. Coast. Shelf Sci.* **55**, 547–555 (2002).

34. Archer, S. D. *et al.* Processes That Contribute to Decreased Dimethyl Sulfide Production in Response to Ocean Acidification in Subtropical Waters. *Front. Mar. Sci.* **5**, 1–19 (2018).

35. Taucher, J. *et al.* Influence of Ocean Acidification and Deep Water Upwelling on Oligotrophic Plankton Communities in the Subtropical North Atlantic: Insights from an In situ Mesocosm Study. *Front. Mar. Sci.* **4**, 1–18 (2017).

36. Moore, C. M. *et al.* Processes and patterns of oceanic nutrient limitation. *Nat. Geosci.* **6**, 701–710 (2013).

37. McParland, E. L. & Levine, N. M. The role of differential DMSP production and community composition in predicting variability of global surface DMSP concentrations. *Limnol. Oceanogr.* **64**, 757–773 (2019).

38. Bürgermeister, S. *et al.* On the biogenic origin of dimethylsulfide: relation between chlorophyll, ATP, organismic DMSP, phytoplankton species, and DMS distribution in Atlantic surface water and atmosphere. *J. Geophys. Res.* **95**, 607–615 (1990).
